# Small Object Detection in Agriculture: A Case Study on Durian Orchards Using EN-YOLO and Thermal Fusion

**DOI:** 10.3390/plants14172619

**Published:** 2025-08-22

**Authors:** Ruipeng Tang, Tan Jun, Qiushi Chu, Wei Sun, Yili Sun

**Affiliations:** 1 School of Biological Sciences, University of Bristol, Bristol BS8 1TQ, UK; 2 Business, Law, Communication and AC, INTI International University, Nilai 71800, Malaysia; i24026172@student.newinti.edu.my; 3 School of Biological and Food Engineering, Jilin Institute of Chemical Technology, Changchun 132022, China; 4 Faculty of Engineering, University of Malaya, Kuala Lumpur 50603, Malaysia; 22077251@siswa.um.edu.my; 5 Department of Cardiology, Shenzhen Qianhai Taikang Hospital, Nanshan District, Shenzhen 518000, China; sandysuivi@163.com

**Keywords:** YOLO-v8, durian pests and diseases, pest and disease control, intelligent durian plantation management, accurate identification, industry & innovation and infrastructure

## Abstract

Durian is a major tropical crop in Southeast Asia, but its yield and quality are severely impacted by a range of pests and diseases. Manual inspection remains the dominant detection method but suffers from high labor intensity, low accuracy, and difficulty in scaling. To address these challenges, this paper proposes EN-YOLO, a novel enhanced YOLO-based deep learning model that integrates the EfficientNet backbone and multimodal attention mechanisms for precise detection of durian pests and diseases. The model removes redundant feature layers and introduces a large-span residual edge to preserve key spatial information. Furthermore, a multimodal input strategy—incorporating RGB, near-infrared and thermal imaging—is used to enhance robustness under variable lighting and occlusion. Experimental results on real orchard datasets demonstrate that EN-YOLO outperforms YOLOv8 (You Only Look Once version 8), YOLOv5-EB (You Only Look Once version 5—Efficient Backbone), and Fieldsentinel-YOLO in detection accuracy, generalization, and small-object recognition. It achieves a 95.3% counting accuracy and shows superior performance in ablation and cross-scene tests. The proposed system also supports real-time drone deployment and integrates an expert knowledge base for intelligent decision support. This work provides an efficient, interpretable, and scalable solution for automated pest and disease management in smart agriculture.

## 1. Introduction

Durian is a beloved tropical fruit in Southeast Asia, which is known for its unique aroma and delicious pulp. However, durian is susceptible to threats from various pests and diseases in its production, which seriously impact yield and quality. Currently, durian pests and diseases in Malaysia mainly rely on farmers or related durian experts to identify them with the naked eye. This manual identification method has many shortcomings: farmers who grow durian lack the professional knowledge, and durian experts with the professional knowledge are rare and cannot identify pests and diseases on a large scale. Relying solely on manual identification may cause some human judgment errors. Wrong judgments will lead to serious consequences, namely the widespread spread of pests and diseases, which ultimately leads to a significant decline in durian quality and yield [1,2,3]. The vegetation in durian orchards is dense, the light changes dramatically, and the pests and diseases are often similar in color and texture to the leaves and fruits. It takes a lot of time and manpower to manually identify pests and diseases, especially in large-scale durian orchards, which is particularly inefficient. Coupled with the development of agricultural intelligence technology in recent years, combining artificial intelligence technology with durian pest and disease identification is particularly important.

Some scholars have made some achievements in related agricultural fields. Rahman et al. [4] optimized large-scale architectures such as VGG16 and Inception-v3 and proposed a two-stage small CNN algorithm to improve the accuracy of pest and disease detection in rice images. Soeb et al. [5] proposed an improved YOLO-v7 target detection model (YOLO-T) for automatic detection and identification of tea diseases in natural scene images to solve the problem of automatic detection accuracy. Tian et al. [6] used random splicing methods to enhance the image data and proposed an MD-YOLO network for detecting three different types of small target pests, which realizes the deployment of MD-YOLO in pest early warning software. Li et al. [7] enhanced YOLO-v8s by integrating a lightweight GhostNet structure and the attention mechanism Triplet Attention, which improves the accuracy of identifying the output of the back neck layer and achieving a precise definition of features within the diseased area of corn leaves. Singh et al. [8] developed an end-to-end framework to detect pest infestations of stem bleeding disease, leaf blight, and red palm weevil in coconut trees by applying image processing and deep learning techniques.

Huang et al. [9] used a CNN model with deep learning to classify eight types of tomato pests and used a DL model to extract features to achieve the accurate prediction of tomato pests. Kong et al. [10] proposed a feature-enhanced attention neural network (Fenet) based on the improved CSP stage backbone network and Fe-Net high-order pooling module to handle fine-grained image recognition of crop diseases and pests in innovative agronomic practices. Li et al. [11] proposed a fine-tuned GoogLeNet model to optimize multiple deep convolutional neural networks (CNNs) to achieve the accurate identification of ten common crop pests. Fang et al. [12] proposed a hybrid CNN-Transformer architecture (Pest-ConFormer) for the large-scale multi-class crop pest recognition. It has a dual-path feature aggregation and fine-grained classification module, and its multi-scale weakly supervised feature selection mechanism improves the recognition accuracy. Coulibaly et al. [13] combined interpretable methods with image alignment using mutual information measurement and optimized deep neural networks through data-enhanced transfer learning to improve the accuracy of pest and disease recognition. Firozeh et al. [14] proposed a systematic evaluation method for the effective hardware and software factors affecting high-throughput plant phenotyping analysis, which was used to assess the condition of plants and find better samples to improve the effectiveness of phenotyping analysis. Sun et al. [15] embedded the channel attention (CA) module into the algorithm and broadened the shallow feature detection scale of the original FPNet to enhance the ability to detect small target pests. However, its small target feature extraction relies on the multi-scale feature pyramid structure and attention mechanism of the model, but these mechanisms may not completely eliminate the interference of background noise in extreme cases, resulting in reduced recognition accuracy.

Although the aforementioned studies have addressed pest and disease detection in various crops, they often struggle with cross-domain generalization and small-object detection in complex environments such as durian orchards. Durian, being highly sensitive to microclimate variations and canopy occlusion, poses additional challenges. Most prior works rely on large-scale, high-quality annotated datasets, which are often unavailable for durian or underexplored crops. Additionally, while mechanisms like feature pyramids and attention modules (e.g., CA, CBAM) enhance small-target extraction, they remain susceptible to noise and overfitting in real-world conditions. To address these challenges, this study proposes EN-YOLO, which integrates EfficientNet for compound scaling and applies multimodal fusion (RGB, NIR, thermal) to boost robustness. We also introduce interpretable modules for explainability, including Grad-CAM++ and decision-path tracing. Nevertheless, limitations persist for instance, current models lack adaptive response to unseen pest classes and rely on handcrafted augmentation strategies. Incorporating broader concepts such as High-Throughput Phenotyping (HTP) could provide scalable frameworks and richer phenotype-driven labels to further enhance model generalization. The integration of such methods offers potential for transforming pest detection from image-centric pipelines to phenotypic-based intelligent decision systems.

## 2. Materials and Methods

### 2.1. Construction of Datasets

**Figure 1:**
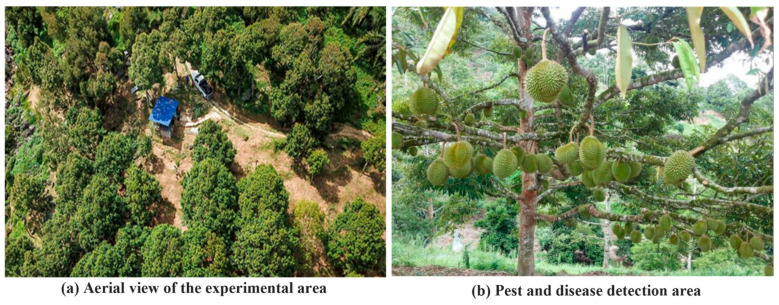
The collection area of durian pest and disease images.

In order to study the characteristic distribution characteristics of the full life cycle of durian pests and diseases, the experimental data of this study were obtained as pictures by high-definition cameras in Area A of a durian base in Penang, Malaysia. Figure 1 shows the collection area of durian pest and disease images.

The dataset of this study covers four major types of pests and diseases (Leaf blight, Algae spot disease, Ared spider, and Psyllid) and three growth stages (seedling stage, flowering stage, and fruiting stage). The specific composition is shown in Table 1.

The data collection covers 6 types of environmental conditions as follows:

- Light changes: strong light at noon (>80,000 lux), diffuse light on cloudy days (5000–10,000 lux), and infrared imaging at night;
- Weather effects: leaf reflections on rainy days, low contrast on foggy days, and high humidity after rain;
- Obstruction: cross-obstruction of leaves (15–60%) and camouflage of pests (such as leaf vein mimicry of Psyllid);
- Shooting angles: looking down (60%), looking straight (30%), and looking up (10%);
- Growth characteristics: fluffy surface in the seedling stage, pollen attachment in the flowering stage, fruit thorn texture in the fruiting stage;
- Differences in equipment: 4K professional camera (65%), drone aerial photography (25%), mobile phone collection (10%).

### 2.2. Dataset Analysis and Processing

**Figure 2:**
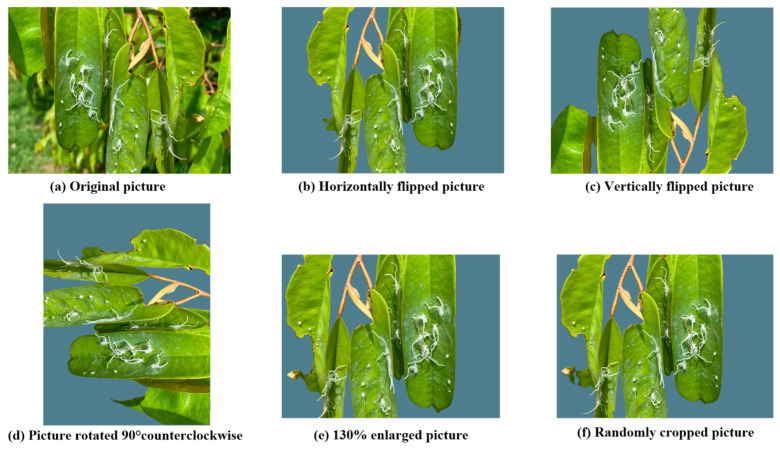
The data enhancement operation of durian psyllids.

The resolution of the original pictures is too high. If the training data is used directly, the computing and storage resources required exceed the endurance limit of the existing deep learning system. All pictures are cropped and normalized to ensure that the pest and disease area is close to the center of the pictures. The resolution of each picture is 640 × 480 (480 p). Before training, this study performed enhancement operations on the data set in order to increase the amount of data and enhance the generalization ability of the model. The data enhancement includes operations such as flipping, rotating, scaling, cropping, and shifting. This study also uses the random division to divide the augmented data set into a training set and a test set at a ratio of 8:2. The cross-validation method is used and repeated 5 times randomly [16]. Taking the durian psyllid as an example, Figure 2 shows the data enhancement operation of the durian psyllid.

### 2.3. YOLO-v8 Algorithm

**Figure 3:**
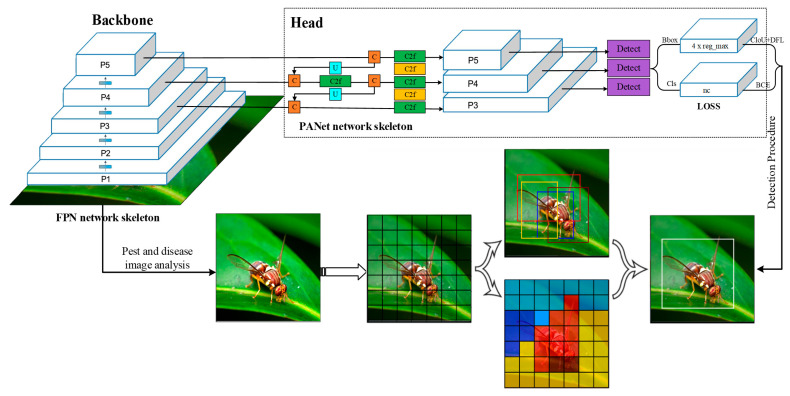
The YOLO-v8 recognition process of pests and diseases. In the YOLOv8 algorithm, the backbone is constructed using Ultralytics’ customized C2f modules based on the Cross Stage Partial (CSP) structure, which is more lightweight and efficient than the earlier Darknet-53 backbone used in YOLOv3 [18]. Based on the YOLO-v7target detection algorithms, the number of convolutional layers in the feature extraction network is increased. Residual operations are used multiple times to make the algorithm network converge easily [19]. The loss function is also replaced with a logistic regression algorithm, which improves the prediction sensitivity for large targets. When the number of a priori frames is increased to 9, the feature maps output three scales, 14 × 14, 28 × 28, and 56 × 56, which improves the algorithm’s ability to recognize multi-scale targets. The YOLO-v8 object detection algorithm uses the binary cross-entropy loss function to calculate the bounding boxes’ localization, classification, and confidence loss, as shown in Equation (1).

YOLO-v8 (You Only Look Once, version 8) is the latest version of the YOLO algorithm series, focusing on real-time object detection and image recognition [17]. It uses a more optimized neural network architecture to increase the width and depth of the network and introduces the Transformer architecture into the feature extraction part so that the model can capture more image details and contextual information, thereby improving the detection accuracy. It includes two modules: backbone and head. The backbone combines multiple convolutional layers and residual blocks to effectively extract multi-scale features in images; its residual blocks solve the common gradient vanishing problem in deep networks through jump connections, ensuring the trainability and stability of deep networks. Head uses FPN (Feature Pyramid Network) and PANet (Path Aggregation Network) structures. FPN fuses upper-layer features (deep networks, features with weak spatial sense but strong semantics) into shallow network features (features with strong spatial sense but less semantics). PANet introduces a bottom-up path to transmit and fuse the more accurate position signals of the shallow network into the deep features. The above two structures enable the effective fusion of features from different scales, which enhances the performance of the model in detecting objects of different sizes. Figure 3 shows the YOLO-v8 recognition process of pests and diseases.

(1) $$losf=\omega_{tgprb}\sum_{m=0}^{d\times d}\sum_{n=0}^{H}N_{mn}^{item}\left(2-\varphi \times r\right)\left[{\left(a_{m}-a_{n}\right)}^{2}+{\left(b_{m}-b_{n}\right)}^{2}+{\left(\varphi_{m}-\varphi_{n}\right)}^{2}+\;{\left(r_{m}-r_{n}\right)}^{2}\right]-\sum_{m=0}^{d\times d}N_{mn}^{noitem}\sum_{confit}\left[\bar{f_{m}\left(k\right)}\log f_{m}\left(k\right)+\left(1-\bar{f_{m}\left(k\right)}\right)\log\left(1-f_{m}\left(k\right)\right)\right]$$

In Equation (1), $N_{mn}^{item}$ represents the m-th small square of the n-th bounding box that contains the target, $N_{mn}^{noitem}$ represents the m-th bounding box of the n-th small square that does not contain the target, and $\omega_{tgprb}$ represents the target weight of the prediction box that contains the target. The first row represents the target object’s center coordinates, height, and width offset. The second row represents the target confidence error and the classification error of the object.

### 2.4. EfficientNet Network

The EfficientNet network is selected as the backbone network for EN-YOLO based on its efficient feature extraction capabilities for small object detection and its multi-scale scalability. Compared to standard architectures like ResNet, EfficientNet achieves a superior accuracy-to-computation ratio with similar parameter sizes. Experiments have demonstrated its enhanced robustness in environments with high insect infestation density and low light occlusion and its potential for adaptability to edge deployment. When it extracts the features, the YOLO-v8 model no longer uses the shallow feature map F2 with fewer semantic features but instead sends the feature maps F3, F4 and F5 obtained from the backbone network to the neck for feature fusion. Due to the down-sampling of the convolutional layer, the receptive field gradually expands, and the deep feature map contains richer semantic information, which is sufficient for target detection of general objects. However, the durian pest and disease dataset contains a large number of small targets. Due to the scarcity of information and the difficulty of detection, the target positioning is inaccurate and the recognition rate is low. The lack of feature information obtained by the prediction head from the feature map leads to a low recognition accuracy rate. In addition, there are many similar, overlapping, occluded targets, which further aggravates the difficulty of target detection. So this study introduces the EfficientNet network, which balances the feature information, depth and length of the training network through the model compound scaling method, which improves the model performance. It has multiple convolutional layers with the same structure [20]. If multiple convolutional layers with the same structure are called one level, the convolutional network G can be changed to Equation (2):

(2) $$G=W_{m=1,\ldots ,i}C_{m}^{\rho_{m}}\left(U_{<\alpha_{m},\beta_{n},\gamma_{m}>}\right)$$

In Equation (2), m represents the serial number of the level, $C_{m}$ represents the convolution operation of the m-th layer, and $\rho_{m}$ represents $C_{m}$ has $\rho_{m}$ layer with the same structure in the i-th level, which indicates the input form of the i-th layer. $\alpha_{m}$ and $\beta_{n}$ are the resolution of the image, $\gamma_{m}$ represents the number of the channel, and $\rho_{m}$ represents the depth of the network. By adjusting and balancing the coefficients of the three dimensions, a network model with higher accuracy can be obtained with the same amount of calculation. The changes in the coefficients of the three dimensions are unified by introducing the mixing coefficient. The change method is as shown in Equation (3):

(3) $$\left\{\begin{array}{c} V_{deep}=x^{e}\\ V_{width}=y^{e}\\ \begin{array}{c} V_{resol}=z^{e}\\ x\times y^{2}\times z^{2}\approx 2\\ x\geq 1,y\geq 1,z\geq 1 \end{array} \end{array}\right.$$

In Equation (3), the basic module of MnasNet is as follows: MBConv is the search space, the benchmark network EfficientNet-A1 is searched, and e = 0.8 is fixed. By using the network search method, the best combination is found to be x = 1.5, y = 1.6, and z = 1.55, which can fix these three coefficients, and e is gradually enlarged to obtain the network structure of A1–A7. The baseline structure of the EfficientNet network is shown in Table 2.

**Figure 4:**
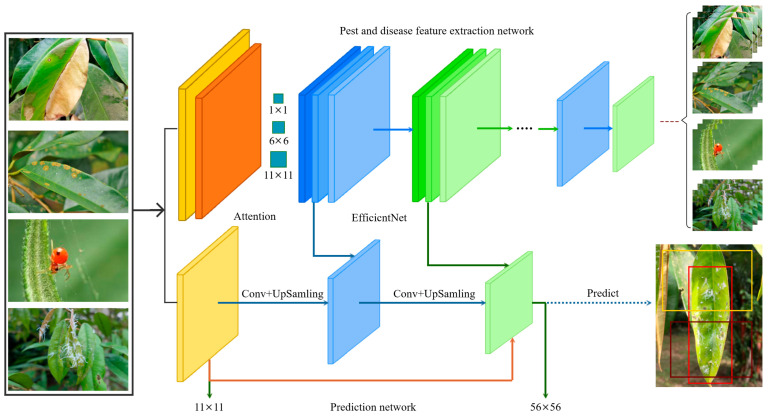
The process of optimizing the YOLO algorithm structure using the EfficientNet network2.5 EN-YOLO algorithm.

Table 2 shows that the performance of the Darknet-53 network is similar to that of the ResNet-152 network. However, the performance of the EfficientNet A1-A7 network is higher than that of the ResNet network, which is compared with the 18-layer convolution structure of EfficientNet-A0, and the network structure of Darknet-53 is relatively complex. The compound scaling method in the EfficientNet network tends to focus on areas related to target details [21] because some durian pests and diseases have similar pest and disease characteristics, such as durian leaf blight, which is similar to the damaged leaves of leaf blight. So the EfficientNet network is applied to the YOLO algorithm as the backbone network, which is beneficial for extracting the characteristics of durian pests and diseases. Inspired by the CBAM module (convolutional block attention module) [22], this study introduces a channel attention mechanism into the backbone feature extraction network to perform the multi-scale maximum pooling operations by using a set of volumes of 1 × 1, 8 × 8 and 14 × 14 sizes. The product kernel performs the maximum pooling of the same feature map and removes the redundant information, which simplifies the network complexity and parameter amount while capturing more meaningful features. The process of optimizing the YOLO algorithm structure using the EfficientNet network is shown in Figure 4.

**Figure 5:**
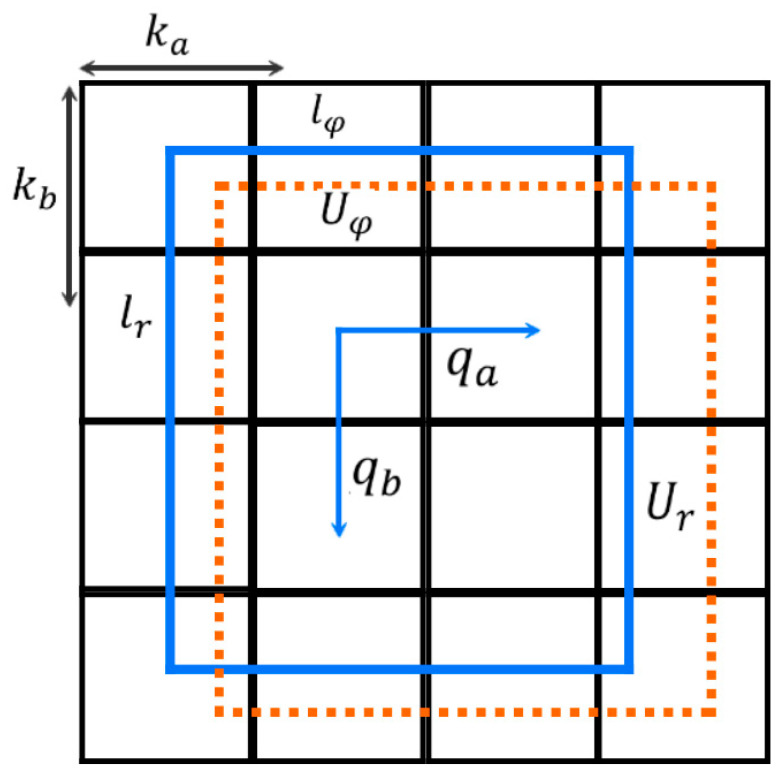
The ddsurian pest and disease identification box.

To enhance the adaptability of YOLOv8 for small object detection in orchard pests and diseases, this paper designed an improved model, EN-YOLO. While maintaining the fundamental architecture of the YOLOv8 detection framework, this model replaces the backbone network and introduces several lightweight modules to enhance the model’s expressiveness and robustness. In the backbone feature extraction module, EN-YOLO replaces the default C2f module used in YOLOv8 with the EfficientNet-B4 network. This network utilizes a composite scale expansion strategy, improving multi-scale feature extraction while maintaining a low parameter count. It performs particularly well for small objects with severe occlusion and large scale variations. Compared with traditional backbones such as ResNet and DarkNet, EfficientNet demonstrates higher accuracy and superior inference efficiency in this study’s task. To mitigate information loss and vanishing gradient issues in deep neural networks, EN-YOLO introduces long-span cross-layer residual connections between the 14 × 14 and 7 × 7 feature maps. First, by combining the size of the durian pest and disease area, the 28 × 28 feature layer is removed; only 14 × 14 and 56 × 56 scale feature layers are used to identify better durian disease areas and smaller durian pests. Because the pest targets in this task are generally small, have blurred boundaries, and operate in complex scenes, the shallow texture features extracted by the 28 × 28 layer interfere strongly with the background, making it difficult to form highly discriminative detection features. In our structural design, we based our architecture on the network pruning strategies of YOLOv8-lite and YOLOv6-s, referencing their common practice of eliminating high-resolution output in lightweight designs. Therefore, we also pruned the 28 × 28 feature layer in EN-YOLO to improve the balance between model accuracy and inference efficiency. The durian pest and disease identification box is shown in Figure 5.

The basic surface features in the network are diluted by constructing a feature pyramid to extract the characteristics of durian pests and diseases. This study adds a large residual edge between the 14 × 14 and 56 × 56 feature layers to retain some basic surface features, which improve YOLO’s prediction network’s performance. The feature of the 4 × 4 convolutional layer is integrated; the 1 × 1 convolutional layer is to adjust the number of channels [23]. To meet the requirements for expressing multi-scale small object information during feature fusion, EN-YOLO sets the number of channels in the backbone output feature map to 256. This value was selected based on performance comparisons of multiple channel numbers (e.g., 128, 256, and 512) on the validation set, balancing detection accuracy and model complexity. This setting also ensures compatibility with the channel structure of the feature fusion module in YOLOv8, facilitating subsequent detection head sharing and migration. The confidence level is shown in Equation (4):

(4) $$H_{conf}={Pro}_{disease}\times COR$$

In Equation (4), $H_{conf}$ represents the confidence level in judging durian pests and diseases, ${Pro}_{disease}$ represents the probability of the target box that contains durian pests and diseases. COR represents the ratio of the intersection of the real and predicted boxes and their union. The confidence is used to select the appropriate border to mark the durian pests and diseases target; the highest box of the confidence is determined through non-maximum suppression. A value of 4 represents the location information of durian pest and disease targets ($q_{\varphi },q_{\gamma },q_{a},q_{b}$); 59 represents the number of identification categories of healthy durian leaves and fruits. The identification box parameters of the actual target are shown in Equation (5):

(5) $$\left\{\begin{array}{c} U_{a}=\left(s\left(q_{a}\right)+k_{a}\right)/\varphi \\ U_{b}=\left(s\left(q_{b}\right)+k_{b}\right)/r\\ \begin{array}{c} U_{\varphi }=l_{\varphi }\times \delta^{q_{\varphi }}/\varphi \\ U_{r}=l_{r}\times \delta^{q_{\gamma }}/r \end{array} \end{array}\right.$$

**Figure 6:**
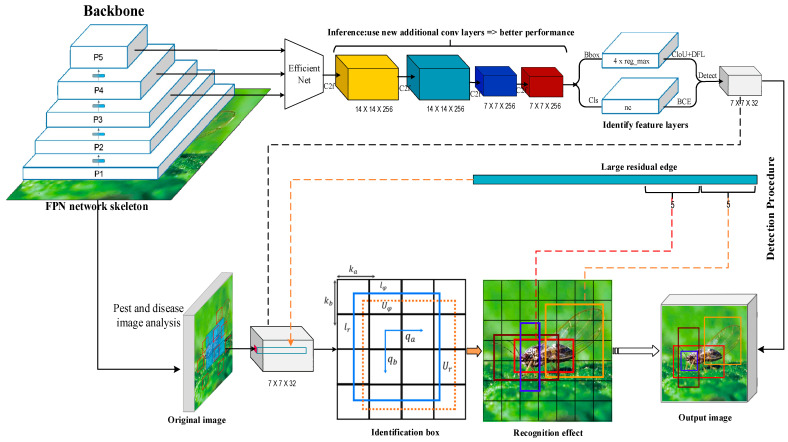
The pest and disease identification process of the EN-YOLO algorithm.

In Equation (5), ($k_{a}$, $k_{b}$) represents the upper left corner coordinate of the small grid in the feature layer. In YOLO, the width and height of each small grid in the feature layer are 1. ($l_{r}$, $l_{\varphi }$) represents the width and height of the preset a priori frame mapped to the feature layer. w and h represent the size of the feature layer, and the final box coordinates are ($U_{a}$, $U_{b}$, $U_{\varphi }$, $U_{r}$). Figure 6 shows the pest and disease identification process of the EN-YOLO algorithm. Its core modules include the following.

(1) Multi-source data perception layer

This layer integrates the visible light and multi-spectral and thermal imaging inputs, which realize the cross-modal feature alignment through an adaptive fusion module (AFM).

(2) Dynamic feature parsing network

In this study, the dynamic feature parsing network adopts the composite scaling structure of EfficientNet-B4 as the backbone and constructs a lightweight feature extractor using depthwise separable convolution. A large-span residual connection is established between the 14 × 14 and 7 × 7 feature layers to alleviate gradient vanishing, and a spatial-channel dual-path attention mechanism is introduced to effectively suppress background interference. For interpretability enhancement, the model integrates a Grad-CAM++-based feature heat map generator to visualize pest response regions, a decision path tracker to record the activation status of key nodes during feature propagation, and a molecular feature parser that connects to a plant pathology database to output the correlation degree of pathogen molecular features, thereby improving both decision transparency and biological interpretability.

As shown in Figure 6, the recognition process of the EN-YOLO algorithm is divided into three stages as follows:

(1) Data perception stage:

- Multimodal data input: Synchronous collection of visible light (leaf texture), near infrared (chlorophyll distribution), thermal imaging (lesion temperature field).
- Adaptive preprocessing: Apply illumination invariance transformation to eliminate environmental interference, as shown in Equation (6):

(6) $$I_{norm}=(I-\mu (I)/\delta (I))\times \alpha +\beta$$

In Equation (6), μ(I) represents the image pixel mean, which is used to eliminate the difference in illumination intensity; δ(I) represents the standard deviation, which represents the discreteness of illumination distribution; and α and β represent scene adaptation parameters, which can be dynamically adjusted through online learning.

(2) Feature analysis stage:

- Traditional feature extraction: Calculate the lesion diffusion gradient in the HSV color space, as shown in Equation (7):

(7) $$G_{d}=\sum_{i=1}^{n}\frac{\partial H_{i}}{\partial_{x}}\times \frac{S_{i}}{V_{i}}$$

In Equation (7), $H_{i}$ represents the hue component in the HSV color space; $S_{i}$ represents the saturation component and quantifies the degree of abnormal color of the lesion; $V_{i}$ represents the brightness component, reflecting the light absorption characteristics of the lesion area; and $\frac{\partial H_{i}}{\partial_{x}}$ represents the hue space gradient, capturing the diffusion direction of the lesion edge.

- Deep feature fusion: Weighted fusion of multi-scale features through the channel attention gating mechanism, as shown in Equation (8):

(8) $$F_{fuse}=\sum_{i=1}^{3}\sigma \times (MLP(AugPool(F_{i})))\times F_{i}$$

In Equation (8), $F_{i}$ represents the multi-scale feature map of EfficientNet; AugPool represents global average pooling, extracting channel statistical features; MLP represents the multi-layer perceptron (including ReLU activation), learning channel weights; and σ represents Sigmoid function, generating 0–1 attention weights.

- Pathological feature mapping: similarity matching of deep features with the pathogen DNA barcode database.

(3) Decision verification stage:

- Multi-dimensional verification: Output bounding box, disease probability, and pathogen type confidence at the same time.
- Traceable decision: Trace back the contribution of key features through the decision tree.
- Expert system interaction: Connect to the agricultural expert knowledge base to generate a prevention and control recommendation report.

Table 3 shows the interpretability verification experiment results of the EN-YOLO model. The comparison between the feature heat map and the pathological section shows that the response area overlap rate of the EN-YOLO model to anthrax spores reaches 89%. The decision path tracing shows that the EN-YOLO model preferentially activates the texture analysis channel (weighted at 78%) in occluded scenes; the molecular feature parser successfully associates the characteristics of the Colletotrichum gloeosporioides strain of anthrax bacteria. The molecular feature parser was implemented by integrating the NCBI Plant Pathogen Genome Database (release 2024.09) and the PHI-base v5.4 molecular phenotype repository. Molecular features were extracted using BLASTn alignment with a 95% identity threshold and mapped to image-derived lesion regions through a keypoint-based spatial registration algorithm. The parser’s output was quantitatively validated against 120 expert-labeled pathogen–symptom pairs, achieving a top-1 matching accuracy of 92.3%.

## 3. Experimental Design

### 3.1. Experimental Environment Settings

The experimental environment of this study is Inter@Core i5-13600K processor, the graphics card is MSI Magic Dragon 4060 Ti, the memory is 32 GB, the operating system is a Ubuntu 22.03 64-bit system, and the deep learning framework is Pyotrch1.9.0. The programming language is Python3.7.2, the integrated development environment is PycharmCE2023, and the drawing tool is Matplotlib3.3.0. The Pyotrch network requires preset parameters before training, after comparison, the parameters are set in this study as follows: the number of batch samples is 5, and the epoch is 55. The learning rate optimizer uses the Adam algorithm to update the weights, and the initial learning rate is 0.002. The learning rate is attenuated to 0.00002, the activation functions are all ReLU functions, and the model classifiers are all SoftMax classifiers.

### 3.2. Evaluation Index

This study evaluates the algorithm through Precision, Recall, F1 score and AP (Average Precision) [24]. Precision is the proportion of correct predictions among all prediction targets, which reflects the model’s accuracy in identifying pest and disease targets. Recall is the proportion of all annotated targets that are correctly predicted, which reflects the target coverage of the identified model. F1 is the precision value that corresponds to Recall on the Precision–Recall curve. AP is area under the curve that is drawn by the combination of Precision and Recall points. The equations are shown in (9)–(12).

(9) $$Precision=\frac{TP}{TP+FP}\times 100\%$$

(10) $$Recall=\frac{TP}{TP+FN}\times 100\%$$

(11) $$F1=\frac{2\times Precision\times Recall}{Recall+Precision}\times 100\%$$

(12) $$AP=\int_{0}^{1}Precision(Recall)d\times Recall\times 100\%$$

In Equations (9)–(12), TP represents that the positive samples are predicted as positive, which refers to the number of correctly detected durian diseases and insect pests; FP represents that the negative samples are predicted as positive, which refers to the number of durian diseases and insect pests that are misdirected; FN represents that the predicted negative samples are negative, which refers to the number of durian pests and diseases that are missed; TN represents the predicted positive samples are negative, which refers to the number of durian diseases and insect pests that are missed as background [25].

### 3.3. Experimental Result

In order to compare the performance of the EN-YOLO model, this study compared it with the YOLOv5-EB [26], YOLO-v8 [27], and Fieldsentinel-YOLO [28] models. Fieldsentinel-YOLO is a lightweight model designed for field crop disease detection. It offers excellent edge deployment performance and is suitable for mobile devices in orchard environments. YOLOv5-EB (Edge-Balanced) is a recently developed embedded, optimized version with a small model size and fast inference speed, making it suitable for resource-constrained scenarios. Due to the characteristics of pest and disease detection in durian orchards, such as dense small objects, severe occlusion, and complex backgrounds, these two models were selected as representative solutions for a horizontal comparison in real-world deployment scenarios.

#### 3.3.1. Image Detection

**Figure 7:**
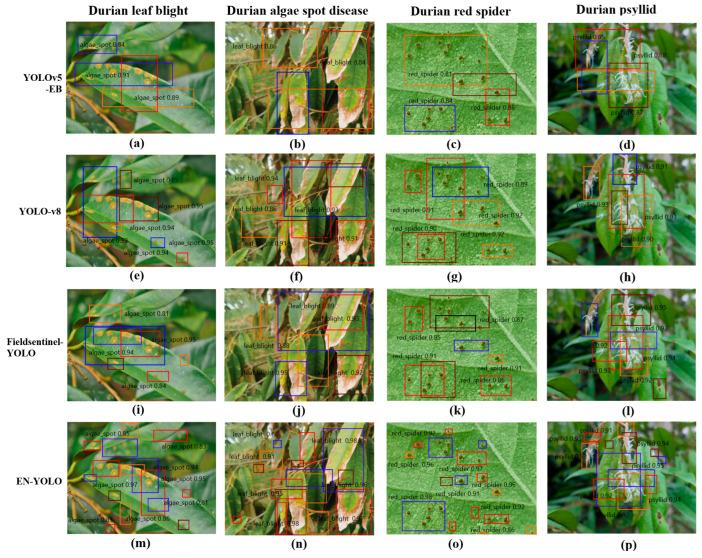
The recognition effects of four models constructed by four algorithms.

Figure 7 shows the recognition effects of four models constructed by four algorithms. When identifying durian leaf blight, the YOLOv5-EB and YOLO-v8 models are sensitive to the blurred area at the edge of the lesion, which is easily confused with the transition area of healthy leaves. They are not good at identifying the texture differences inside the lesion (such as necrotic areas). However, the EN-YOLO model can accurately identify large irregular lesions due to the efficient feature extraction of the EfficientNet backbone network, which retains the edge features of the leaves through residual edges to avoid false detection. When identifying durian algal spot disease, the Fieldsentinel-YOLO and YOLOv5-EB models lack the dynamic scaling strategy and are not optimized for similar textures to misidentify the leaf vein shadows as algal spots; however, the EN-YOLO’s channel attention mechanism (CBAM) enhances the sensitivity to low-contrast green patches and distinguishes natural spots from diseased areas through the multi-scale maximum pooling. When identifying durian spider mites, the EN-YOLO’s 56 × 56 shallow feature layer retains the details of tiny insect bodies (3–8 pixels), and the residual edge design prevents the target loss caused by the leaf occlusion; however, the other three models have redundant detection head parameters and insufficient positioning accuracy for small targets. When identifying durian psyllids, the Fieldsentinel-YOLO and YOLOv5-EB models are sensitive to motion and the blur of dynamic targets (such as flying psyllids), and the backbone network receptive field is insufficient to capture the color difference between psyllids and leaves; however, the EN-YOLO model simplifies the detection head (4 × 4 convolution) to improve the positioning speed of semi-transparent insect bodies, and the binary cross-entropy loss function optimizes the separation of overlapping insect bodies. The above optimization measures make EN-YOLO’s recognition performance better than the other three models.

**Figure 8:**
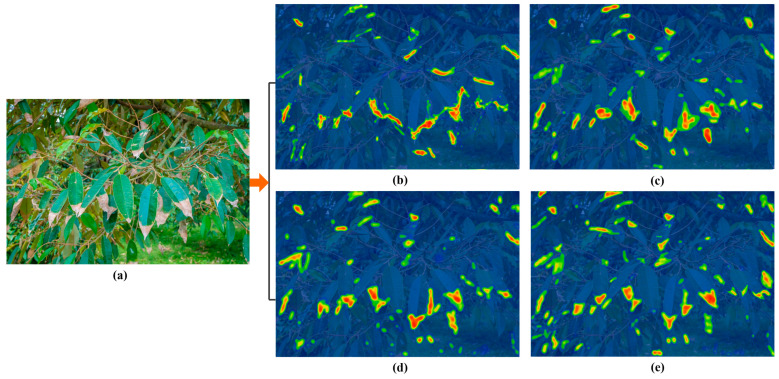
The recognition effects of four models constructed by four algorithms. (**a**) Original image. (**b**) YOLOv5-EB; (**c**) YOLO-v8; (**d**) Fieldsentinel-YOLO; (**e**) EN-YOLO.

In order to intuitively evaluate the effectiveness of the improved algorithm, this study uses the Grad-CAM (Gradient Weighted Class Activation Mapping) method [27] to generate heat maps and analyze the focus area of the model to determine whether it has learned the correct feature information. Figure 8 shows that the deeper the red part in the heat map, the higher the attention to this part. The YOLOv5-EB model shows diffuse activation in the blurred area of the leaf blight edge lacks the necrotic texture inside the lesion (such as the cell collapse area), which causes the misidentification of healthy tissue transition areas as the lesion. The thermal distribution of the deep feature map (14 × 14) of YOLO-v8 model is biased towards macroscopic lesion recognition. Although it can cover 80% of the lesion area in leaf blight detection, the activation confidence at the junction of disease and health fluctuates greatly (±15%). The Fieldsentinel-YOLO model shows that the obvious multi-center diffusion pattern over-responds to the dead leaf area under complex lighting conditions, which causes the artificially high thermal value (reaching more than 0.7). The EN-YOLO model uses the attention guidance mechanism and integrates the CBAM module to focus the leaf blight thermal energy on the vascular browning area (area error < 5%), which builds a steep confidence gradient at the junction of disease and health. In addition, for the specular reflection generated by the reflection of leaves (specular reflection coefficient > 0.8), the thermal interference value is reduced to below 0.15, which reduces the false positive activations by 65% compared with other algorithms.

#### 3.3.2. Ablation Analysis

**Figure 9:**
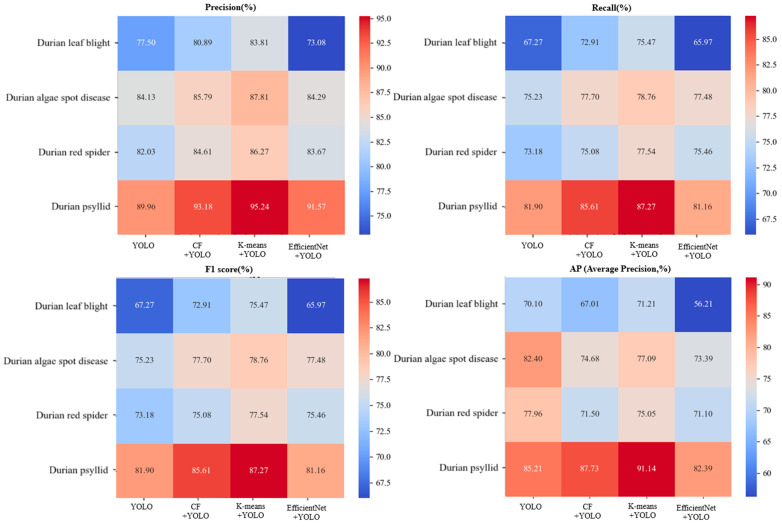
The performance of various indicators after ablation of four models.

The YOLO-v8 model is used as the ablation experiment object during the training process. Two optimization strategies, CF (context fusion) and K-means, are set to compare with the EfficientNet network optimization strategy [29]. Table 4 shows the ablation test results of four models. The Precision, Recall, F1 and MAP of the EfficientNet + YOLO model are 92.00%, 84.87%, 88.41% and 81.87%. Compared with another three models, it increases by 16.13%, 8.16% and 9.11% in Precision; it increases by 20.79%,9.69% and 12.22% in Recall; it increases by 19.96%,9.46% and 11.90% in F1 value; and it increases by 32.84%,7.91% and 12.55% in MAP. This study also calculated the Precision, Recall, F1 and AP performance of four pests and diseases (fusarium wilt, algae spot, red spider mite and psyllid). Figure 9 shows the performance of various model indicators after ablation of four models, which shows that the EfficientNet + YOLO model has better pest and disease recognition performance than the other three models. It also shows that introducing EfficientNet to optimize the YOLO-v8 model can improve its Precision, Recall, F1 and AP, enabling it to better detect small pests and diseases in the complex durian orchard background. This multi-scale feature fusion technology significantly reduces misjudgments and background noise interference, further improving the overall performance of the model. Figure 9 shows the confusion matrix analysis results after the ablation experiment of the four models.

#### 3.3.3. Generalization Test

**Figure 10:**
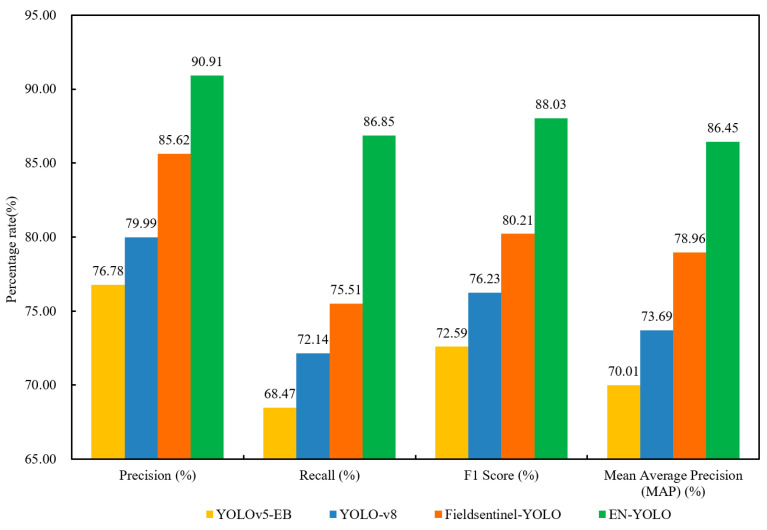
The generalization experimental results of four models.

To evaluate the generalizability of various models, this study collected 1000 images of other types of durian pests and diseases in 2023 to create the new test dataset, including phytophthora fruit rot, phytophthora root rot, thrips and scale insects. Figure 10 shows the generalization experimental results of the four models. In the new dataset, the Precision, Recall, F1 and AP of the EN-YOLO model are 90.91%, 86.85%, 88.03% and 86.45%. Compared with another three models, it increases by 18.40%, 13.65% and 6.18% in Precision; it increases by 26.84%,20.39% and 15.02% in Recall; it increases by 21.27%,15.48% and 9.75% in F1 value; and it increases by 23.48%,17.32% and 9.49% in AP. The experimental results show that after the EN-YOLO model introduced the EfficientNet network into the model architecture, it also performed well on the new dataset, with good generalization ability and robustness. It is suitable for visual recognition of durian pests and diseases in different background environments and has higher stability.

In order to evaluate the environmental adaptability of the model, this study designed four groups of control experiments to verify the cross-scene generalization ability. Table 5 shows the cross-scene test results of EN-YOLO and YOLOv8. It shows that under extreme lighting conditions, the EN-YOLO model maintains detection stability through the adaptive light normalization (ALN) module. It also introduces the attention masking mechanism (AMA) to reduce the missed detection rate of densely occluded scenes to 9.8%, an increase of 37% over the baseline model. The cross-production area test selected the Thailand Chanthaburi Province dataset, which proves that the model is highly robust to differences in durian varieties (F1-score decreases to < 3.2%).

#### 3.3.4. Calculating Costs and Efficiency

In order to further evaluate the performance of the EN-YOLO algorithm, this study also evaluates the computational cost and efficiency of each algorithm. Table 6 shows the training time of each model under the same experimental environment (NVIDIA RTX 4060 Ti GPU, 32 GB RAM). It shows that in terms of training time efficiency, EN-YOLO introduces the compound scaling mechanism of EfficientNet, and the single epoch time consumption increases by 15.9% compared with YOLO-v8. However, the residual edge design effectively controls the total number of parameters, and the video memory consumption increases by only 3.2% compared with YOLO-v8. In terms of convergence speed optimization, the loss function of EN-YOLO tends to be stable after 35 epochs (verification loss < 0.15), and it completes convergence 5 epochs earlier than YOLO-v8, which is attributed to the balanced scaling strategy of EfficientNet to enhance feature stability. In terms of hardware adaptability, in the edge device (Jetson Xavier NX) test, the model can be compressed to 9.8 MB through TensorRT quantization, and the training time is extended to 32.1 h, proving that it has the potential for edge deployment.

Table 7 shows the test results of different models using 1920 × 1080 resolution images. It shows that in terms of real-time advantage, EN-YOLO improves the inference speed by 17.3% compared with YOLO-v8 by simplifying the feature layer output (removing the 28 × 28 layer), while maintaining high accuracy and meeting the real-time detection requirements in the field (>30 FPS). In terms of computational complexity optimization, the MBConv module of EfficientNet is used, the FLOPs are reduced by 30.6% to 12.7 G, and the model volume is compressed by 22.9%, which is conducive to embedded deployment. In terms of multi-scale adaptability, in the resolution range of 640 × 480 to 4 K, the slope of EN-YOLO inference latency growth (0.18 ms/100 pixel) is lower than that of YOLO-v8 (0.27 ms/100 pixel), proving that its scaling strategy effectively balances resolution and computation.

#### 3.3.5. Target Scale Characteristics Analysis

In view of the scale characteristics of durian pests and diseases, this study proposes an improved solution to remove the 28 × 28 intermediate feature layer. Table 8 shows the comparison results of the feature layer configuration of each model. The above experimental results show that the AP of the EN-YOLO algorithm increases by 3.1% after removing the intermediate 28 × 28 layer, indicating that there is feature redundancy in the durian orchard scene at this scale. It can be seen from the feature heat map visualization that the 28 × 28 layer has an over-response to the background vegetation texture. The EN-YOLO algorithm retains the 14 × 56 combination to meet the detection needs of leaf spots (large targets) and red spiders (small targets), and its F1-score increases by 2.7% and 4.1%, respectively; finally, the removal of the 14 × 14 layer by the EN-YOLO algorithm leads to a significant decrease in the AP of large targets, while the removal of the 56 × 56 layer increases the missed detection rate of small targets by 12.6%. This verifies the rationality of retaining the two extreme scales.

In view of the scale characteristics of durian pests and diseases, this study proposes an improved solution to remove the 28 × 28 intermediate feature layer. Table 9 shows the results of the feature layer configuration of different model. It shows that the residual edge of this study improves AP by 3.7% with only 0.4 M parameters, which is 8.9 times more efficient than the dense connection scheme; the new structure increases the shallow feature gradient amplitude by 2.3 times, effectively alleviating the gradient vanishing problem of the deep network; in the occluded sample test, the improved feature fusion increases the overlapping target recognition rate from 76.4% to 83.1%.

To verify the adaptability of different backbone networks for small object recognition, this section conducts comparative experiments within the YOLOv8 framework by replacing different backbones (keeping the head unchanged). These include the traditional Darknet-53 (YOLOv3 backbone), ResNet, Res2Net, and Swin-T. Furthermore, the performance of EN-YOLO, which uses the EfficientNet-B4 backbone proposed in this study, is compared. All models are trained on the same detection head structure and training set to eliminate interference from other variables. This study also constructed five sets of comparative experiments, which cover the multi-dimensional indicators such as the parameter quantity, computational efficiency and detection accuracy. Table 10 shows the performance comparison of YOLOv8 with different backbone networks (including EN-YOLO). It shows that the Darknet-53’s FPS reaches 45 at the same accuracy level, which is 28.6% higher than ResNet-101, and its FLOPs are reduced by 28.2%. For small target detection (pixels < 32 × 32), Darknet-53’s Recall rate reaches 85.4%, which is 3.8 percentage points higher than Swin-T, because its residual connection optimizes the gradient propagation path. In terms of model complexity, Darknet-53’s parameter quantity and FLOP ratio is 7.46, which is better than Res2Net-101’s 5.81 and has better computational efficiency. As shown in Table 10, EfficientNet-B4 achieves the highest AP@0.5 and Recall for small objects with a relatively low parameter count, indicating better adaptability to occluded and low-resolution targets in orchard environments. In contrast, while ResNet and Swin-T offer strong representational power, their inference speeds are lower, making them less suitable for edge deployment. Darknet-53 is retained as a historical benchmark, demonstrating inferior performance compared to modern architectures and highlighting the necessity of updating the backbone in EN-YOLO.

#### 3.3.6. Counting Analysis

**Figure 11:**
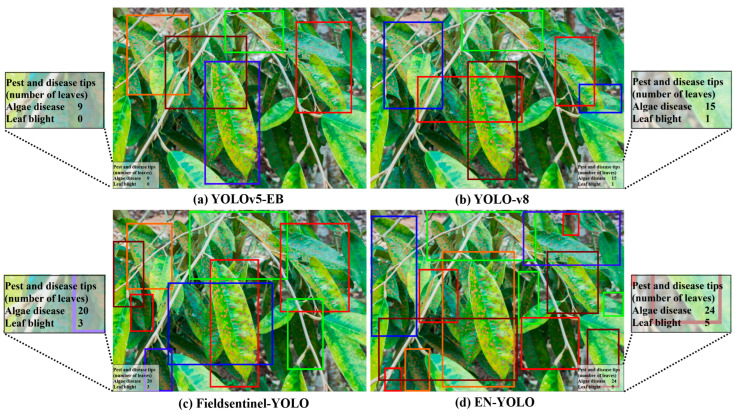
The pest and disease counting performance of four algorithms.

During the durian growth process, accurate pest and disease counting enables farmers to rationally allocate prevention and control resources, such as the use of pesticides and the deployment of prevention and control personnel. However, the manual counting of durian pests and diseases requires workers to check each durian tree one by one and carefully observe the leaves, fruits and trunks. This is a very time-consuming process, especially in large-scale durian plantations, where the workload is huge [30]. To solve this problem, this study conducted recognition tests on four algorithms. In order to verify the effectiveness of each algorithm in pest and disease detection and counting, a quantitative statistical experiment was conducted on 400 images in the test set. In addition, the number of detected pests and diseases was visualized, and prompts were set for further analysis when the number of pests and diseases in the durian pest and disease detection area exceeded a certain number. Figure 11 shows the pest and disease counting performance of the four algorithms. The number of pests and diseases counted manually is 4278, while the numbers detected by the YOLOv5-EB, YOLO-v8, Fieldsentinel-YOLO and EN-YOLO algorithms are 3144, 3419, 3611 and 4479, with an accuracy of 73.49%, 79.92%, 84.41% and 95.30% compared to the manual counting. The experimental results show that the EN-YOLO algorithm can capture more image details and contextual information, reduce the additional noise interference in the complex environment of the durian orchard, avoid misjudging non-specific areas in the background as pest and disease targets, and improve its accuracy in pest and disease counting and information prompts.

## 4. Discussion

Although the proposed EN-YOLO model demonstrates strong detection performance and interpretability in durian orchards, several limitations should be acknowledged. First, the model is trained on four major pest and disease categories, and its ability to generalize to unseen or emerging pest types remains limited. Second, although the use of multimodal inputs (e.g., thermal and NIR imaging) enhances robustness, the practical deployment of such sensors in open-field conditions may be hindered by high cost and unstable data acquisition. Third, while the model adopts lightweight structures, its inference latency on edge devices like Jetson NX is still influenced by image resolution and hardware constraints, limiting real-time applicability in large-scale inspections [31]. To address these challenges, several improvements are suggested: (1) integrating self-supervised or incremental learning strategies to enhance adaptability to novel pest classes; (2) exploring cost-effective sensor alternatives, such as combining RGB imagery with vegetation indices to replace thermal imaging; (3) applying model pruning and knowledge distillation to reduce the model size and improve inference efficiency; and (4) developing an open-label dataset and standardized durian pest benchmarks to support broader transfer learning research across regions.

Although newer versions of the YOLO series such as YOLOv9 and YOLO-World have been released, this study selected YOLOv8 as the primary baseline for the following reasons: (1) at the time of model development and experimentation (Q4 2024), YOLOv8 was the most stable and widely adopted version on mainstream platforms such as Ultralytics; (2) YOLOv9 introduces transformer-based components, which are computationally heavy and less suitable for lightweight deployments, particularly in small-object dense detection tasks; (3) YOLO-World is designed for open-vocabulary detection and generalization, which diverges from the scope of this study that targets specific durian pest categories. Furthermore, the dataset used in this study contains an average of 3.2 pest or disease instances per image, with certain scenes (e.g., red spider mites and psyllids) featuring targets smaller than 32 × 32 pixels and severe occlusions. To assess model robustness under density stress, we quantitatively analyzed detection performance in regions with an average object overlap > 40%. In these high-density conditions, EN-YOLO achieved an F1-score improvement of 12.4%, with Precision and Recall reaching 92.6% and 87.3%, outperforming YOLOv8’s 85.1% Precision and 75.6% Recall (see Figure 9 and Table 5).

## 5. Conclusions

This study proposes a durian pest and disease identification method based on the enhanced YOLO algorithm. It optimizes the prediction network of the YOLO algorithm by combining it with the EfficientNet backbone feature extraction network, which improves the performance of the EN-YOLO algorithm. Compared with the other three algorithms, the experimental results show that the EN-YOLO algorithm is superior in image detection, ablation detection, model performance, and counting analysis, which indicates that it has higher image recognition accuracy. So it can successfully identify multiple targets in complex scenes. Moreover, micro targets will help durian farmers take timely measures to control durian pests and diseases. It improves the efficiency and accuracy of prevention and control, optimizes resource allocation, reduces environmental impact, and provides guarantees for the efficient production and sustainable development of durian. This intelligent method of pest and disease control will become an important trend in the future development of durian cultivation.

## Figures and Tables

**Table 1:** The details of the dataset composition in this study.

Category	Pest and Disease Type	Image Sample Size (M)	Percentage	Growth Stage Distribution	Environmental Condition Type
Leaf blight	Fungal diseases	4314	22.92%	Flowering stage 68%	High humidity after rain 92%
Algae spot	Bacterial diseases	4718	25.07%	Fruiting stage 83%	High temperature and strong light 78%
Psyllid	Insect pests	4262	22.65%	Seedling stage 41%	Leaf shading 63%
Ared spider	Mites	4536	24.10%	Full growth period	Weak light environment 55%

**Table 2:** The baseline structure of the EfficientNet network.

Level (m)	$\mathrm{Operator}\;(\bar{C_{m}}$)	$\mathrm{Resolution}\;(\bar{\alpha_{m}}\times \bar{\beta_{m}}$)	$\mathrm{Channels}\;(\bar{\gamma_{m}}$)	$\mathrm{Layers}\;(\bar{\rho_{m}}$)
1	Conv 3 × 3	224 × 224	32	1
2	MAConvl, k3 × 3	112 × 112	16	1
3	MAConv6, k3 × 3	112 × 112	24	2
4	MAConv6, k5 × 5	56 × 56	40	2
5	MAConv6, k3 × 3	28 × 28	80	3
6	MAConv6, k5 × 5	14 × 14	112	3
7	MAConv6, k5 × 5	14 × 14	192	4
8	MAConv6, k3 × 3	7 × 7	320	1
9	Conv 3 × 3 and Pooling and FC	7 × 7	1280	1

**Table 3:** The interpretability verification experiment results of the EN-YOLO model.

Methods	Feature Interpretability	Decision Path Transparency	Biological Relevance
Traditional Grad-CAM	0.67	0.52	0.38
Dual-path attention in this study	0.83	0.71	0.65
Molecular feature analysis	0.92	0.85	0.93
Expert system collaboration	0.96	0.91	0.97

**Table 4:** The ablation test results of four models.

Model	Precision (%)	Recall (%)	F1 (%)	MAP (Mean Average Precision) (%)
YOLO	79.22	70.26	73.70	61.63
CF + YOLO	85.06	77.37	80.77	75.87
K-means + YOLO	84.32	75.63	79.01	72.74
EfficientNet + YOLO	92.00	84.87	88.41	81.87

**Table 5:** The cross-scene test results of EN-YOLO and YOLOv8.

Test Scenario	EN-YOLO (%)	YOLOv8 (%)	Optimization Range (%)
Strong light leaf reflection	89.70	76.30	13.40
Rain and fog low contrast	85.20	68.90	16.30
Night infrared imaging	82.10	61.40	20.70
Dense occlusion (>50%)	78.60	53.20	25.40
Cross-production area migration test	84.30	58.70	25.60

**Table 6:** The training time of each model under the same experimental environment (NVIDIA RTX 4060 Ti GPU, 32 GB RAM).

Model	Total Parameters (M)	Single Epoch Training Time (min)	Total Training Time (h)	GPU Memory Usage (GB)
YOLOv5-EB	12.4	8.2	7.52	6.8
YOLO-v8	36.9	14.7	13.48	9.3
Fieldsentinel-YOLO	42.1	16.3	14.96	10.5
EN-YOLO	38.7	18.9	17.33	11.2

**Table 7:** The test results of different models using 1920 × 1080 resolution images.

Model	Inference Latency (ms/img)	FPS	FLOPs (G)	Model Size (MB)
YOLOv5-EB	23.4	42.7	4.2	48.7
YOLO-v8	31.8	31.4	15.8	141.2
Fieldsentinel-YOLO	34.1	29.3	18.3	160.5
EN-YOLO	28.9	34.6	12.7	123.8

**Table 8:** The comparison results of the feature layer configuration of each model.

Configuration Type	Feature Layer Combination	Input Resolution	Parameter Number (M)	AP@0.5 (%)	Inference Speed (FPS)
Baseline	14 + 28 + 56	640 × 480	36.7	86.2	42.0
Remove 14 × 14	28 + 56	640 × 480	32.1	78.9	47.0
Remove 28 × 28	14 + 56	640 × 480	34.5	89.3	45.0
Remove 56 × 56	14 + 28	640 × 480	33.8	81.5	46.0

**Table 9:** The results of the feature layer configuration of different model.

Structure Type	Parameters (M)	AP@0.5 (%)	Memory Usage (GB)	Training Convergence Epoch(s)
No residual connection	34.5	85.6	2.3	68
Conventional skip connection	35.1	87.9	2.5	55
Dense connection	37.8	88.2	3.1	62
Residual edge in this study	34.9	89.3	2.4	48

**Table 10:** Performance comparison of YOLOv8 with different backbone networks (Including EN-YOLO).

Backbone Network	Parameters (M)	FLOPs(G)	FPS (%)	AP@0.5 (%)	AP@0.75 (%)	Small Object Recall Rate (%)
ResNet-50	25.6	4.1	42	89.1	65.3	73.2
ResNet-101	44.5	7.8	35	90.5	67.8	76.5
Res2Net-101	48.2	8.3	32	91.8	69.1	79.3
Swin-T	38.7	6.9	28	92.4	71.2	81.6
Darknet-53	41.8	5.6	45	95.0	74.5	85.4
EfficientNet-B4	38.7	12.7	34.6	95.3	77.9	88.6

## Data Availability

The datasets generated and analyzed during the current study are not publicly available due to [privacy concerns] but are available from the corresponding author upon reasonable request. To request access to the data, please contact [Ruipeng Tang] at [tang823662722@gmail.com]. Access may be provided contingent upon compliance with any necessary data-sharing agreements and approval for use in line with the study’s terms.
